# Hedgehog Signal Inhibitor GANT61 Inhibits the Malignant Behavior of Undifferentiated Hepatocellular Carcinoma Cells by Targeting Non-Canonical GLI Signaling

**DOI:** 10.3390/ijms21093126

**Published:** 2020-04-28

**Authors:** Kensuke Harada, Ryuya Ohashi, Kyoko Naito, Keita Kanki

**Affiliations:** Department of Biomedical Engineering, Faculty of Engineering, Okayama University of Science, 1-1 Ridai-cho, Kita-ku, Okayama 700-0005, Japan; t15s047hk@ous.jp (K.H.); t16s011or@ous.jp (R.O.); t20mm02nk@ous.jp (K.N.)

**Keywords:** GANT61, non-canonical Hedgehog-GLI signaling, hepatocellular carcinoma

## Abstract

The Hedgehog (HH)–GLI pathway plays an important role in cell dedifferentiation and is therefore pivotally involved in the malignant transformation of cancer cells. GANT61, a selective inhibitor of GLI1 and GLI2, was reported as a promising treatment for cancer in various tissues; however, the biological impact of GANT61 in hepatocellular carcinoma (HCC), especially in undifferentiated HCC cells, remains unclear. In this study, we investigated the antitumor effect of GANT61 using two undifferentiated hepatoma cell lines: HLE and HLF. Quantitative PCR and RT-PCR analyses revealed that these cells express *GLI* transcripts, showing mesenchymal phenotypes characterized by the loss of epithelial and hepatic markers and specific expression of epithelial–mesenchymal transition (EMT)-related genes. GANT61 significantly reduced the proliferation and cell viability after drug treatment using 5-FU and Mitomycin C. We showed that *GLI* transcript levels were down-regulated by the MEK inhibitor U0126 and the Raf inhibitor sorafenib, suggesting that non-canonical signaling including the Ras–Raf–MEK–ERK pathway is involved. Sphere formation and migration were significantly decreased by GANT61 treatment, and it is suggested that the underlying molecular mechanisms are the down-regulation of stemness-related genes (*Oct4*, *Bmi1*, *CD44,* and *ALDH*) and the EMT-related gene *Snail1*. The data presented here showed that direct inhibition of GLI might be beneficial for the treatment of dedifferentiated HCC.

## 1. Introduction

Hepatocellular carcinoma (HCC), the most frequently diagnosed primary liver cancer, is an aggressive disease caused by chronic hepatitis B or C virus infection, excessive alcohol intake, non-alcoholic steatohepatitis, or exposure to aflatoxin B [1]. The prognosis for patients with advanced stage HCC is poor because of its high rates of recurrence and intrahepatic metastasis. Consequently, there is an essential requirement for the development of an improved therapeutic strategy that aims to inhibit tumor progression. The malignant transformation of tumor cells is related to dedifferentiation of the cells [2]. A loss of cellular specific functions, increased proliferation, and enhanced glycolytic activity are the common features of tumor cells. Additional genetic mutations often cause aberrant activation of intracellular signaling pathways, leading to a loss of cell–cell adhesion, increased cell motility, and anchorage-independent cell proliferation, which are the crucial malignant phenotypes that trigger invasiveness and metastasis [3].

The Hedgehog (HH) pathway, originally identified as an important pathway during embryonic development, is known to play a role in carcinogenesis in various tissues including the liver [4]. In recent years, inappropriate HH signaling was demonstrated in more than 30% of human cancers [5]. Canonical activation of HH signaling is triggered by the binding of HH ligands (SHH, IHH, and DHH) to their receptor Patched-1 (PTCH) on the surface of target cells. Ligand-bound PTCH lost its inhibitory regulation on Smoothened (SMO); thus, active SMO causes the downstream glioma-associated homologue (GLI) transcription factors GLI1 and GLI2 to be activated via nuclear translocation [6,7]. GLI target genes include factors involved in cell proliferation, survival, self-renewal, and invasiveness; therefore, aberrant activation of this signal pathway has a positive correlation with poor prognosis [8,9]. Small-molecule inhibitors of the HH-GLI signal, such as SMO inhibitors, were developed to increase the clinical benefit of patients. Some have entered clinical trials as promising therapies for advanced cancers, including basal cell carcinoma and medulloblastoma [10,11,12]. However, several patients were reported to be resistant to the SMO inhibitor because of an acquired mutation in SMO that disrupts drug binding and mutations in genes downstream of SMO, such as SUFU mutations [13,14,15]. Moreover, an alternative activation pathway of GLI, namely the non-canonical HH pathway, has been identified [16]. These problems of drug resistance led to the development of new small molecules that function as direct inhibitors of GLI transcription. 

GANT61 is a selective inhibitor of GLI1- and GLI2-mediated gene transactivation. Preclinical studies were performed for numerous cancer types, including rhabdomyosarcoma, neuroblastoma, and leukemia, as well as colon, pancreas, cervix, skin, lung, head-neck, and gastric cancers [5]. Although substantial promising results were reported, the biological impact of GANT61 in HCC, in particular undifferentiated HCC cells, has not been clarified. In this study, we investigated the antitumor effect of GANT61 in undifferentiated HCC cells, which show greater expression of GLI transcription factors than well-differentiated HCC cells. The presented data show that GANT61 significantly reduced cell proliferation and viability after drug treatment. Malignant phenotypes, such as sphere formation and migration, were significantly decreased with reduced expression of stemness-related and epithelial–mesenchymal transition (EMT)-related genes. We also found that the gene expression of *GLI* is regulated, in part, by non-canonical signaling, including the Ras–Raf–MEK–ERK pathway, in these cells. Our data suggest that the application of a direct inhibitor of GLI transcription might be beneficial for the treatment of dedifferentiated HCC.

## 2. Results

### 2.1. Preferential Expression of GLI Genes in Undifferentiated HCC Cell Lines

**Figure 1:**
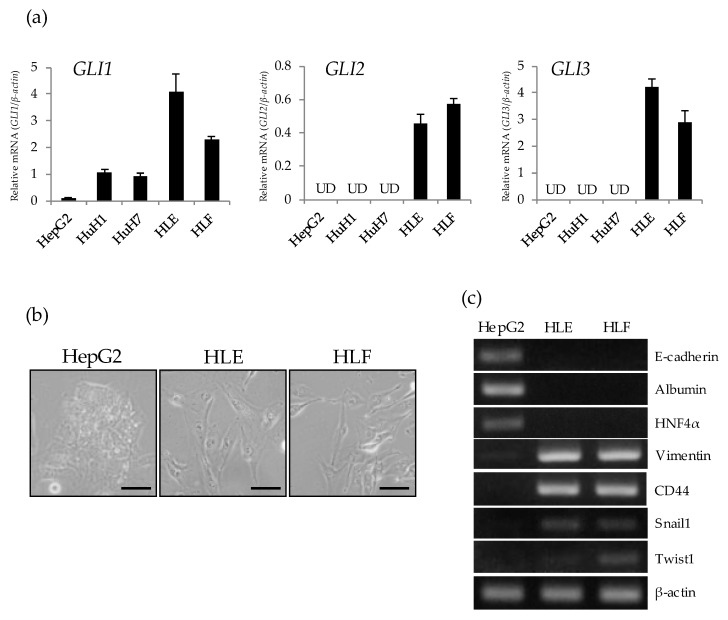
Preferential expression of *GLI* genes in undifferentiated hepatocellular carcinoma (HCC) cell lines. (**a**) Relative gene expression levels of *GLI1*, *GLI2*, and *GLI3* compared to *β-actin* were determined in hepatoma cell lines by qRT-PCR analysis (UD; undetectable). (**b**) Microscopic observation of HepG2 (left), HLE (center), and HLF (right). Scale bar = 20 μm. (**c**) RT-PCR analysis of cell-type specific markers including *E-cadherin* (epithelial), *Albumin*, *HNF4α* (hepatic), *Vimentin*, *CD44*, *Snail1*, *Twist1* (mesenchymal), and *β-actin* as a house keeping gene.

To determine the intracellular status of GLI-mediated signaling in hepatoma cell lines, a gene expression analysis of *GLI1*, *GLI2*, and *GLI3* was performed on a panel of hepatoma cells including three differentiated (HepG2, HuH1, and HuH7) and two undifferentiated (HLE and HLF) types of HCC cell. Quantitative PCR revealed that *GLI1* mRNA is expressed in both differentiated and undifferentiated HCC cells, being highly expressed in undifferentiated cells (Figure 1a). Among HCC cells, HepG2, a typical well-differentiated type of HCC cell showed the lowest expression of the *GLI1* gene. The expression of *GLI2* and *GLI3* was positively detected in undifferentiated cells but not in differentiated HCC cells. Morphologically, HepG2 showed an epithelial-like shape characterized by tight cell adhesion, while HLE and HLF showed mesenchymal morphology characterized by loose cell contact and an irregular cell shape (Figure 1b). RT-PCR analysis revealed that HLE and HLF lack the expression of *E-cadherin* and hepatic markers (*ALB* and *HNF4α*), but these cells express mesenchymal markers such as *vimentin*, *CD44*, *snail1*, and *twist1* (Figure 1c). These data suggest that GLI-mediated signaling is activated in undifferentiated HCC cell lines showing the mesenchymal phenotype. Therefore, we chose HLE and HLF cells to investigate the effect of the HH signaling inhibitor GANT61. 

### 2.2. Antitumor Effect of GANT61 on the Proliferation of Undifferentiated HCC Cells

**Figure 2:**
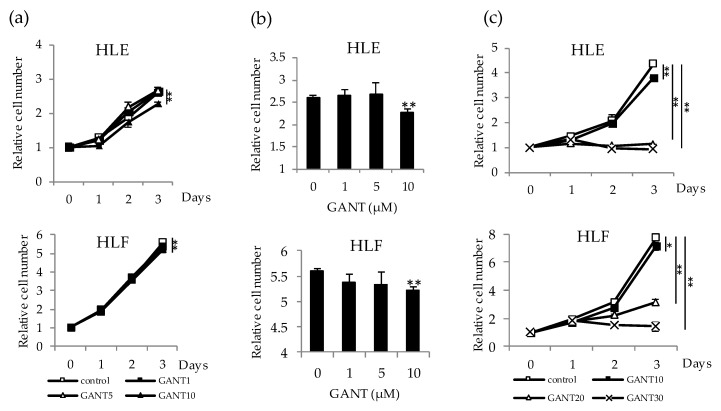
Antitumor effect of GANT61 on the cell proliferation of undifferentiated HCC cells. (**a**) HLE and HLF cells were treated with 0, 1, 5, and 10 μM of GANT61 for 3 days for cell proliferation assays. Cell viability was determined by WST assay and expressed as the relative number of viable cells compared to day 0. (**b**) Column graphs showing the values at day 3 of the experiment. (**c**) HLE and HLF cells were treated with 0, 10, 20, and 30 μM of GANT61 for 3 days and analyzed as described in (a). * *p* < 0.05, ** *p* < 0.01 vs. the vehicle control (Dimethylsulfoxide; DMSO).

To evaluate the antitumor potential of GANT61, cell proliferation was investigated in HLE and HLF cells treated with 0–10 µM of GANT61. No statistically significant effect on cell proliferation was observed during the first two days of treatment (Figure 2a). In both types of cell, significant inhibition of cell proliferation was observed on day 3 of 10 µM treatment (Figure 2b). Treatment with a concentration greater than 10 µM, for example, with 20 or 30 µM, markedly decreased cell viability at days 2 and 3 of the experiment (Figure 2c). Therefore, we selected a treatment with 10 µM of GANT61, a quantity which did not largely affect the viability of HCC cells, for further experiments.

### 2.3. Drug-Specific Enhancing Effect of Cytotoxicity by GANT61 and Involvement of the Ras–Raf–MEK–ERK Pathway in the Regulation of GLI Expression in Undifferentiated HCC Cells

**Figure 3:**
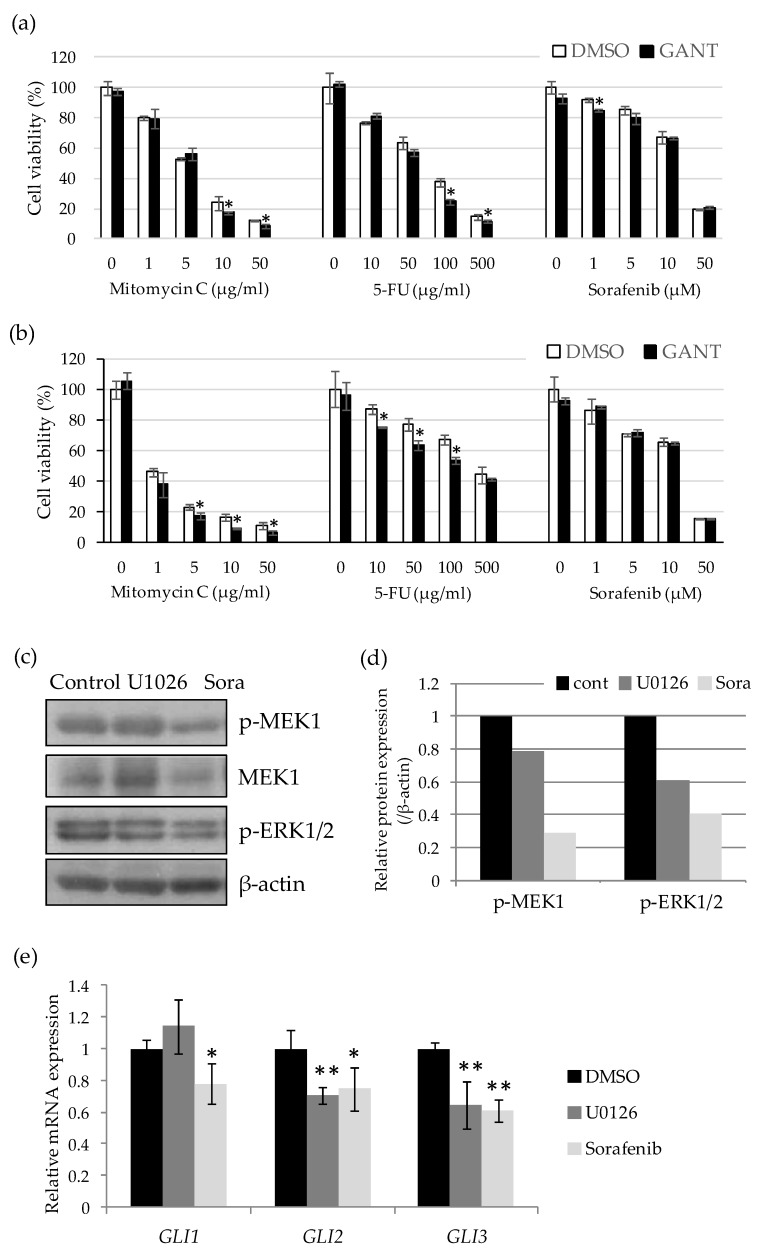
Drug-specific enhancing effect of cytotoxicity by GANT61 and involvement of the Ras–Raf–MEK–ERK pathway in the regulation of *GLI* expression in undifferentiated HCC cells. Cell viability assays of HLE (**a**) and HLF (**b**) cells treated with 10 μM of GANT61 in combination with various concentrations of anticancer drugs. After 48 h, cell viability was measured by WST assay and is expressed as the percentage of drug-free control. (**c**) Western blot analysis of phospho-MEK (Ser2017/221), total MEK, and phospho-ERK (Thr202/Tyr204) in the lysate extracted from the cells treated with U1026 (1 μM) and sorafenib (Sora) (10 μM) for 24 h. β-Actin served as a control of protein loading. (**d**) Relative protein expression levels were calculated from the band intensity with ImageJ software (NIH). (**e**) Relative gene expression levels of *GLI1*, *GLI2*, and *GLI3* in U1026- and sorafenib-treated cells compared to the control (DMSO). * *p* < 0.05, ** *p* < 0.01 vs. the vehicle control (DMSO).

Next, to examine the effect on drug sensitivity, HLE and HLF cells were treated with a series of concentrations of Mitomycin C (0–50 µg/mL), 5-FU (0–500 µg/mL), and sorafenib (0–50 µg/mL) in combination with GANT61 for 48 h. GANT61 was used at 10 µM which showed no inhibitory effect on the proliferation of HLE and HLF cells up to 48 h after the treatment. The three anticancer drugs significantly reduced the cell viability of HLE and HLF cells in a dose-dependent manner; however, the effect of combination treatment with GANT61 differed between the drugs. GANT61 significantly reduced the viability of HCC cells treated with relatively high concentrations of mitomycin C and 5-FU. In contrast, this enhancement of drug sensitivity was barely observed in the combination with sorafenib (Figure 3a,b). To understand the mechanism of the drug-specific effect of GANT61, we next examined the expression level of GLI mRNA in sorafenib-treated cells. Sorafenib is known as a multi-kinase inhibitor that targets the kinase activity of B-Raf, vascular endothelial growth factor receptors (VEGFRs), and platelet-derived growth factor receptor β (PDGFR-β). We first examined the amount of the active forms of MEK1 and ERK1/2, downstream molecules of the Ras–Raf pathway, in HLE cells treated with the inhibitors. Phosphorylated forms of MEK1 (p-MEK1) and ERK1/2 (p-ERK1/2) were shown to be decreased by treatment with the MEK inhibitor U0126 (1 μM) and sorafenib (10 μM) (Figure 3c,d). Quantitative PCR revealed that these treatments significantly decreased the expression levels of *GLI1*, *GLI2*, and *GLI3* mRNA (Figure 3e). Altogether, these data suggest that GANT61 has an antitumor effect by decreasing cell proliferation and cell viability when it is combined with anticancer drugs in undifferentiated HCC cells. The effect of combination treatment on drug sensitivity differed between the drugs due to the non-canonical regulation of GLI-mediated signaling by the Ras–Raf–MEK–ERK pathway, a critical target of sorafenib. 

### 2.4. GANT61 Inhibits the Sphere Forming Capacity of Undifferentiated HCC Cells

**Figure 4:**
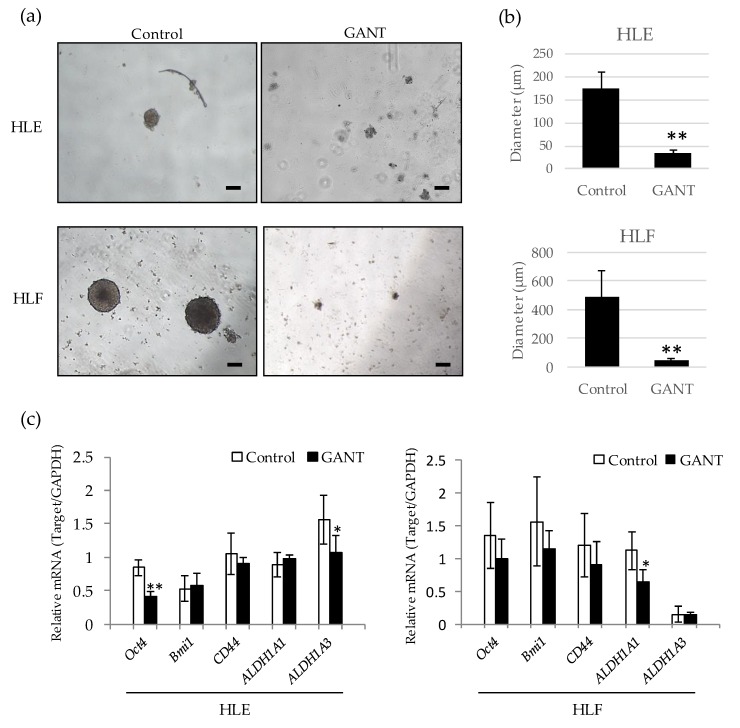
GANT61 inhibits sphere formation by down-regulating stemness-related genes. (**a**) Morphological appearance of spheres formed in the presence or absence of 10 µM GANT61. HLE (upper) and HLF (lower) cells were cultured in an ultra-low-attachment culture plate for 10 days and photographed. Scale bar = 100 µm. (**b**) The diameter of the sphere was measured in five representative spheres of each group. (**c**) Quantitative PCR of stemness-related genes *Oct4*, *Bmi1*, *CD44*, *ALDH1A1*, and *ALDH1A3* in HLE (left) and HLF (right) cells treated with 10 µM of GANT61. *GAPDH* mRNA was used as an internal control. * *p* < 0.05, ** *p* < 0.01 vs. the vehicle control (DMSO).

To examine the effect of GANT61 on the cancer stem cell (CSC)-like phenotype, sphere formation in suspension culture was performed in the presence or absence of GANT61. HLE and HLF cells were able to form large spheres in a low adherence plate with media containing the vehicle. In contrast, large spheres were barely observed in the presence of 10 µM of GANT61 (Figure 4a,b). To understand the possible molecular mechanism underlying this, we next examined the expression of stemness-related genes in control and GANT61-treated cells. Quantitative PCR revealed that *Oct4*, a transcription factor related to pluripotency and self-renewal, was significantly down-regulated in HLE cells treated with GANT61. In HLF cells, GANT61 treatment showed a tendency to reduce the expression of three genes—*Oct4*, *Bmi1* and *CD44*—although the difference was not statistically significant (Figure 4c). Two subtypes of aldehyde dehydrogenase, *ALDH1A1* and *ALDH1A3*, were significantly down-regulated by GANT61 in HLF and HLE cells, respectively. These data suggest that GANT61 inhibits sphere formation by down-regulating stemness-related genes in undifferentiated HCC cells.

### 2.5. GANT61 Inhibits Cell Migration by Down-Regulating Snail1 Gene Expression

**Figure 5:**
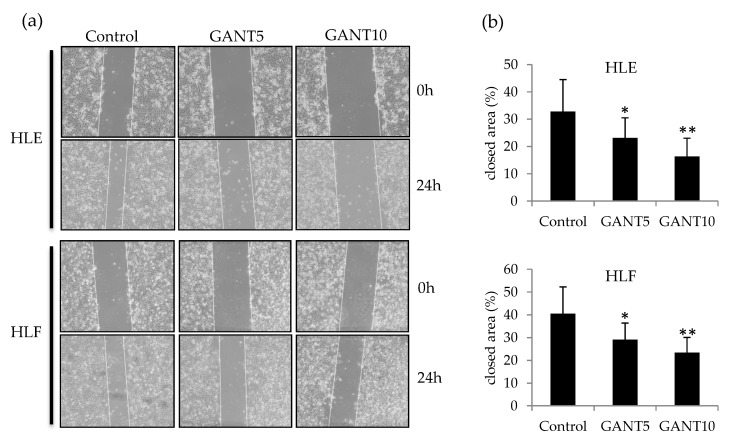
Wound healing assay of undifferentiated HCC cells treated with GANT61. (**a**) Scratched area of monolayer-cultured HLE (upper) and HLF (lower) cells treated with 5 μM (GANT5) and 10 μM (GANT10) of GANT61 under the serum-free condition. (**b**) A closed area was used to evaluate cell migration activity. * *p* < 0.05, ** *p* < 0.01 vs. the vehicle control (DMSO).

To investigate the effect of GANT61 on cell migration ability, a wound healing assay was performed by scratching monolayer-cultured cells. To exclude the effect on cell proliferation, assays were performed under the condition of serum deprivation (1% FBS or serum-free) using 5 and 10 µM of GANT61. Twenty-four hours after scratching, GANT61-treated cells showed slower migration than control cells (Figure 5a). Quantification of cell migrating activity was carried out by measuring a closed area after 24 h, and this revealed a significant and dose-dependent decrease in cell migrating activity in HLE and HLF cells (Figure 5b). 

**Figure 6:**
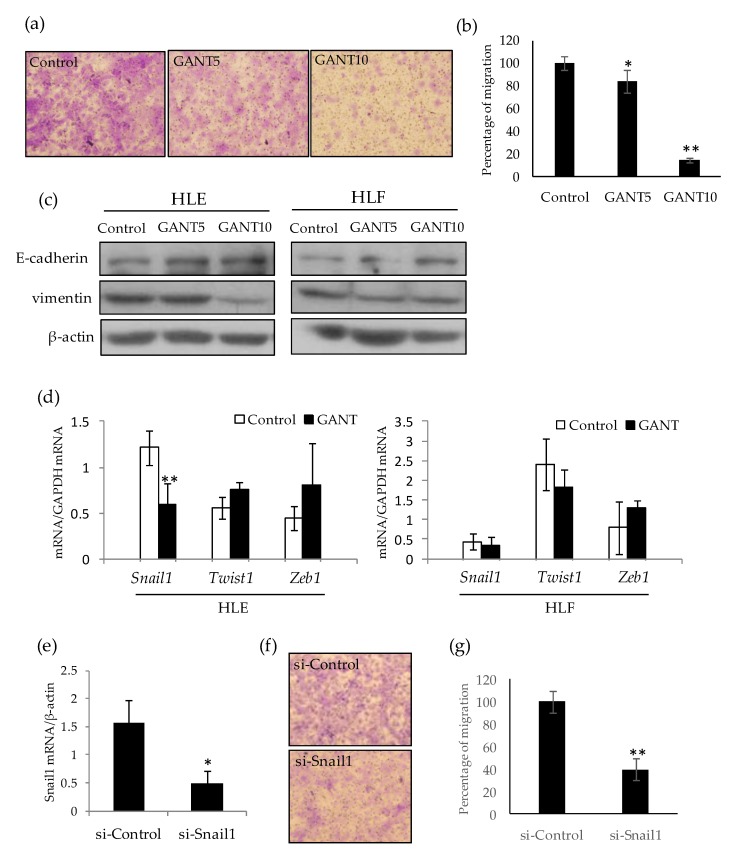
GANT61 inhibits cell migration by attenuating the mesenchymal phenotype of HLE cells. (**a**) Transwell migration assay of HLE cells treated with 5 μM (GANT5) and 10 μM (GANT10) of GANT61. Migrated cells were stained with crystal violet. (**b**) The migration activity of the cells is expressed as a percentage of the cell number compared with the control. (**c**) Western blotting of E-cadherin, vimentin, and β-actin as an loading control. (**d**) Quantitarive PCR analysis of EMT-related transcription factors *Snail1*, *Twist1*, and *Zeb1* in HLE (left) and HLF (right) cells treated with 10 μM GANT61. (**e**) Quantitative PCR analysis of Snail mRNA in HLE cells treated with control (si-Control) and Snail (si-Snail) siRNA. (**f**,**g**) Transwell migration assay of siRNA-treated HLE cells and their migration activity. * *p* < 0.05, ** *p* < 0.01 vs. vehicle control (DMSO).

To confirm the inhibitory effect of GANT61 on cell migration, a transwell assay was performed. As shown in Figure 6, GANT61 lowered the migration of HLE cells in a dose-dependent manner (Figure 6a,b). Furthermore, an increase in the epithelial marker E-cadherin and a decrease in the mesenchymal marker vimentin were observed by Western blotting of the lysates extracted from GANT61-treated cells (Figure 6c). Quantitative PCR revealed that the mesenchymal transcription factor *Snail1* was down-regulated by GANT61 treatment compared to the control (Figure 6d). Snail knockdown by small-interference RNA (siRNA) significantly reduced the migration ability of HLE cells (Figure 6e–g). Together, these data suggest that GANT61 inhibits cell migration by attenuating the mesenchymal phenotype of undifferentiated HCC cells.

## 3. Discussion

The HH-GLI signaling pathway was shown to play a critical function during development by regulating cellular proliferation and differentiation. In wound healing and regeneration processes, this pathway plays important roles in cellular differentiation, migration, and maintenance of tissue progenitor cells [17]. Therefore, aberrant activation of this signal in cancer cells leads to malignant transformations such as dedifferentiation, acquisition of stemness features, and migration potency. In this study, we investigated the biological significance of GLI-mediated signaling in undifferentiated HCC cell lines using the GLI specific inhibitor GANT61. We found preferential expression of *GLI* mRNAs in undifferentiated HCC cell lines that showed the mesenchymal phenotype. Consistent with previous reports, HLE and HLF were characterized by increased mesenchymal markers and the loss of epithelial and hepatic markers [18,19]. Among *GLI* genes, *GLI2* and repressor *GLI3* were specifically expressed in undifferentiated HCC cells. Generally, GLI1 acts mainly transcriptional activation in cancer cells, whereas GLI2 requires N-terminal processing for full activation [20,21]. GLI2 is reported to induce genomic instability by inhibiting apoptosis, which is responsible for eliminating transformed aberrant cells [22]. These reports and our data suggest that *GLI2* expression, in addition to that of *GLI1*, contributes to the promotion of cell dedifferentiation and confers HCC cells with mesenchymal phenotypes.

To determine the biological impact of GANT61 in undifferentiated HCC cells, we investigated the cell proliferation and sensitivity to cell death induced by anticancer drugs in GANT61-treated HLE and HLF cells. Although we observed a positive antitumor effect of GANT61 on cell viability, the impact seemed to be relatively weak compared with that observed in other type of cancers. For instance, previous studies reported that only 10%–40% of cells remained viable after treatment with 10–20 µM of GANT61 in lung, melanoma, prostate, glioma, and breast cancer cells [23,24,25,26,27,28,29]. These reports and our observation allow us to hypothesize that the GANT61 target may be a small subpopulation in HLE and HLF cells. In combination treatment, there was little enhancing effect on drug sensitivity when GANT61was used with low concentrations of mitomycin C and 5-FU; however, a significant decrease in cell viability was observed when GANT61 was used in combination with relatively high concentrations of these drugs. This result suggests that a small population of drug-resistant cells were targeted by GANT61. Previous reports showed that HH-signal inhibitors, such as the SMO inhibitor and direct GLI inhibitors, have antitumor effects by targeting CSC-like cell populations [30,31]. Therefore, it is reasonable to hypothesize that a small subpopulation of CSC-like cells which depends on GLI signaling would be a target of GANT61. 

Interestingly, we observed no enhancement of cell death in combination with sorafenib, a multi-kinase inhibitor targeting the serine–threonine kinases Raf-1 and B-Raf and the receptor tyrosine kinase activity of VEGFRs and PDGFR-β. A possible mechanism underlying the drug-specific response might be explained by the observation that sorafenib down-regulated the expression of *GLI* genes. GLI-dependent transcription is known to be activated in an HH ligand-independent manner, namely non-canonical HH signaling, which includes oncogenic intracellular signaling pathways such as PI3–AKT, TGFβ–smad, and Ras–Raf–MEK–ERK [16]. Therefore, in undifferentiated HCC, the GLI-mediated HH pathway is suggested to be regulated, at least in part, by the Ras–Raf–MEK–ERK pathway.

Increasing evidence is suggesting that the acquisition of stemness features in tumor cells is correlated with poor clinical outcomes in various type of cancers. These malignantly transformed cells often show enhanced resistance to chemotherapy, anchorage-independent survival, and migration potency that causes postoperative recurrence and metastasis. As well as well-known oncogenic intracellular signaling components such as Wnt/β-catenin, Notch, TGF-β, and AKT, HH-GLI signaling was described as an important pathway for maintaining the stemness of undifferentiated and/or immature cells [3]. In normal liver development, HH-GLI signaling contributes to the proliferation of immature progenitor cells, and its activation progressively decreases during liver epithelial cell maturation [32]. HH-GLI signal activation re-increases when the tissue regenerates after injury to promote the proliferation of liver progenitor cells. Therefore, HH-GLI signaling is considered to promote dedifferentiation and confer cancer cells to have stemness features, making it a promising target for cancer therapy. In this study, we observed significant decreases in sphere forming activity and cell migration by GANT61. Gene expression analysis revealed that GLI inhibition significantly down-regulated *Oct4* in HLE cells and in HLF cells, showing a tendency to reduce the expression of stemness-related genes (*Oct4*, *Bmi1*, and *CD44*) which play important roles in anchorage-independent cell growth. We also observed significant down-regulation of *ALDH* mRNA expression by GANT61. ALDH was shown to play an important role in self-renewal in various cancers including HCC and was reported to be regulated by GLI signaling in melanomas [33,34]. Therefore, these stemness-related genes may be crucial targets of GLI signaling in undifferentiated HCC. In the sphere assay, the rate of sphere formation was found to be about 1% for the cells seeded in a low-attachment dish (data not shown). In future research, identification and isolation of a subpopulation that has sphere forming activity is required to assess the critical effect of GANT61. 

A significant decrease in cell motility by GANT61 was confirmed in a wound healing assay and transwell assay. These assays and the Western blot analysis suggest that the mesenchymal features of undifferentiated HCC cells are attenuated by GANT61 treatment. In terms of the molecular mechanism, *Snail1*, a pivotal transcriptional regulator for migration potency, was found to be down-regulated in HLE cells. Although *Snail1* is known as a key regulator of EMT, the impact of GANT61 on mRNA expression differed among two cell lines (Figure 6d). Moreover, GANT61 treatment was associated with greater inhibition of migration activity than *Snail* knockdown, despite the weaker down-regulation effect of *Snail* mRNA compared with that of si-Snail (Figure 6b,g). Together, these results suggest that other genes, in addition to *Snail*, responsible for cell migration may be affected by GANT61. A thorough study of target genes including coding and non-coding genes should be required to understand the cell-type-specific action of GAN61. An anti-migrative effect was related to HH signaling inhibition by the SMO inhibitors NVP-LDE-225 and GDC-0449, both of which were approved for clinical application [35,36,37]. However, these drugs may be ineffective for the non-canonical GLI-activation pathway observed in this study. In addition to the SMO inhibitor, the use of a direct inhibitor of GLI and/or inhibitors of signaling pathways involved in the non-canonical HH-GLI pathway may be required for effective treatment.

**Figure 7:**
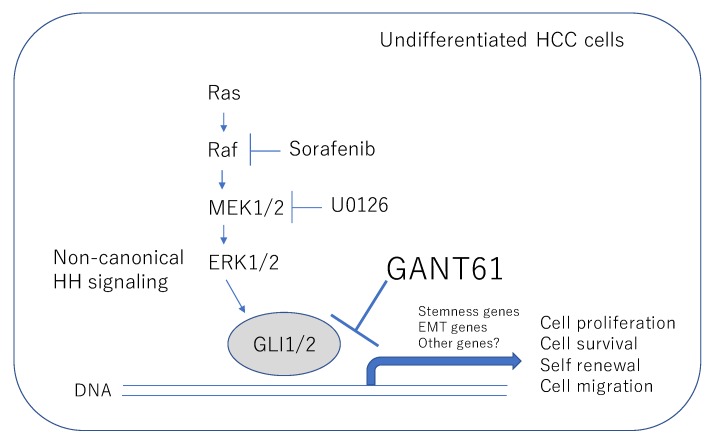
Schematic view of the antitumor effect of GANT61 in undifferentiated HCC cells. GANT61 inhibits the malignant behavior of undifferentiated HCC cells by targeting non-canonical GLI signaling. Arrows denote activation and T-shaped lines indicate inhibition.

As depicted in Figure 7, the results of our study demonstrate that the HH-GLI signal inhibitor GANT61 has an antitumor effect on the malignant character of undifferentiated HCC cells with the mesenchymal phenotype. Direct inhibition of GLI was suggested to be beneficial in the case of dedifferentiated HCC which is involved in non-canonical HH-GLI signaling. 

## 4. Materials and Methods 

### 4.1. Cell Culture and Reagents

Hepatoma cell lines (HepG2, HuH1, HuH7, HLE and HLF) were purchased from RIKEN BRC. Cells were maintained in DMEM containing 10% FBS, 4.5 mg/mL glucose, and 2 mM l-glutamine in a humidified incubator at 37 °C and 5% CO_2_. GANT61 (Santa Cruz Biotechnology; sc-202630) and sorafenib (ChemScene LLC; Monmouth Junction, NJ, USA) were dissolved in DMSO at a concentration of 10 mM and stored in a freezer at –20 °C.

### 4.2. Cell Viability Assays

Cell viability was determined by the WST assay using the Cell Counting Kit-8 (Dojin Chemical Co., Ltd.; Kumamoto, Japan). Briefly, cells were cultured in a 96-well plate and subjected to the experiment. To determine cell viability, a 10% volume of CCK-8 reagent was added to each well, and the plate was incubated for 1 h at 37 °C. Cell viability was calculated by measuring the absorbance at a wavelength of 450 nm. For cell proliferating assays, cells were seeded at 3 × 10^3^ cells/well and measured at 24, 48, and 72 h after the treatment. The relative number of viable cells was calculated from OD450 to create proliferation curves.

### 4.3. Gene Expression Analysis

Total RNA was isolated using the TRIZOL reagent in accordance with the manufacturer's instructions (Invitrogen; Carlsbad, CA, USA). One microgram of RNA was used to synthesize cDNA using GeneAce Reverse Transcriptase (Nippon Gene; Tokyo, Japan). A gene expression analysis was performed using PowerUp SYBR Green Master Mix (Thermo Fisher Scientific; Waltham, MA, USA) and the ABI qPCR system (Thermo Fisher Scientific). The primers used in this study are listed in Supplementary Table S1.

### 4.4. Sphere Forming Assay

A sphere assay was performed by seeding HLE and HLF cells in a 12-well low-attachment plate. Cells were treated with DMSO or 10 μM of GANT61 and incubated for 10 days. Half of the medium was changed every three days. Formed spheres were photographed and their diameters were measured.

### 4.5. Cell Migration Assays

Cells were grown at 90%–100% confluency and subjected to the experiment. Monolayer cells were scratched using microtips and photographed at day 0. Cells were treated with serum-free DMEM containing DMSO or 10 μM of GANT61 for 24 h and photographed at day 1. A closed area was used to evaluate cell migration activity. 

### 4.6. Transwell Migration Assays

HLE cells were pre-treated with DMSO or GANT61 (5 and 10 μM) in a serum-starved condition for 24 h. A total of 5 × 10^4^ cells were seeded into the upper chambers of transwell inserts with 8 μM pores (Corning Inc.; Corning, NY, USA) with DMEM but without serum. The bottom well contained 10% FBS DMEM to induce cell migration. After 24 h, the cells that migrated to the underside of the membrane were fixed and stained with crystal violet containing 10% methanol and counted to quantify the migration ability. A knockdown experiment was performed by transfection of Stealth RNAi Pre-Designed siRNAs (Thermo Fisher Scientific) for *Snail* mRNA and a negative control for 24 h. Cells treated with siRNA were subjected to the transwell assay as described above. 

### 4.7. Western Blot Analysis

Cell lysates were prepared from the cells subjected to the experiment by using Cell Lysis Buffer (Cell Signaling Technology; Danvers, MA, USA) and stored in a deep freezer until use. The protein concentration was determined by the Pierce™ BCA Protein Assay Kit (Thermo Fisher Scientific). Equal amounts of the protein sample were separated on a SDS-PAGE and immunoblotted with antibodies against human E-cadherin (Santa Cruz Biotechnology), vimentin (Santa Cruz Biotechnology), MEK1 (Novus Biologicals, Centennial CO, USA), phospho-MEK1/2 (Ser2017/221) (Cell Signaling Technology), phospho-ERK1/2 (Thr202/Tyr204) (Cell Signaling Technology), and beta-actin (Cell Signaling Technology). HRP-conjugated secondary antibodies were used to detect primary antibodies and were visualized with ECL detection reagent (GE Healthcare UK; Buckinghamshire, UK) and a light-sensitive X-ray film (Fuji film; Tokyo, Japan). 

### 4.8. Statistical Analysis

Statistical comparisons for in vitro experiments were performed using Student’s *t*-tests. *p* < 0.05 was considered statistically significant.

## Supplementary Materials

Supplementary materials can be found at https://www.mdpi.com/1422-0067/21/9/3126/s1.

## Abbreviations

HCC	Hepatocellular carcinoma
HH	Hedgehog
GLI	Glioma-associated homologue
SMO	Smoothened
EMT	Epithelial–mesenchymal transition
RT-PCR	Reverse Transcription-Polymerase Chain Reaction
HNF4α	Hepatocyte nuclear factor 4α
DMSO	Dimethyl sulfoxide
siRNA	Small-interference RNA
ALDH	Aldehyde dehydrogenase
CSC	Cancer Stem Cells
